# Supplementary material for: Heuristics in risky decision-making relate to preferential representation of information
Source: Nat Commun. 2024 May 20;15:4269. doi: 10.1038/s41467-024-48547-z (PMC11106265; doi:10.1038/s41467-024-48547-z)
Supplement: Supplementary file 1 — Supplementary Information [file 41467_2024_48547_MOESM1_ESM.pdf]

Supplementary Information File

Heuristics in risky decision-making relate to preferential representation of information

Evan M. Russek, Rani Moran, Yunzhe Liu, Raymond J. Dolan, Quentin J.M. Huys

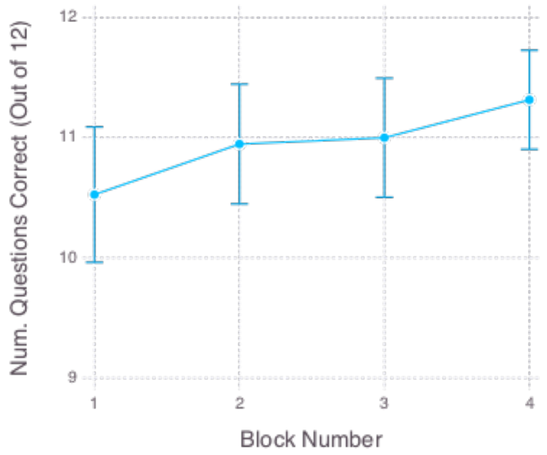

Supplementary Figure 1. Performance on probability quiz. Prior to the decision-making task, yet following the localizer task, participants were trained to learn the probability that each choice stimulus led to each outcome following acceptance (see Methods). Training consisted of 4 blocks. Each block ended with a series of 12 questions, where participants had to answer either which of two outcome stimuli were more likely to follow a choice stimulus (if accepted), or alternatively which of two choice stimuli, if accepted, were more likely to lead to a presented outcome. Line designates mean (+/- s.e.m.) number of questions correct (out of 12) on each block for n=19 participants.

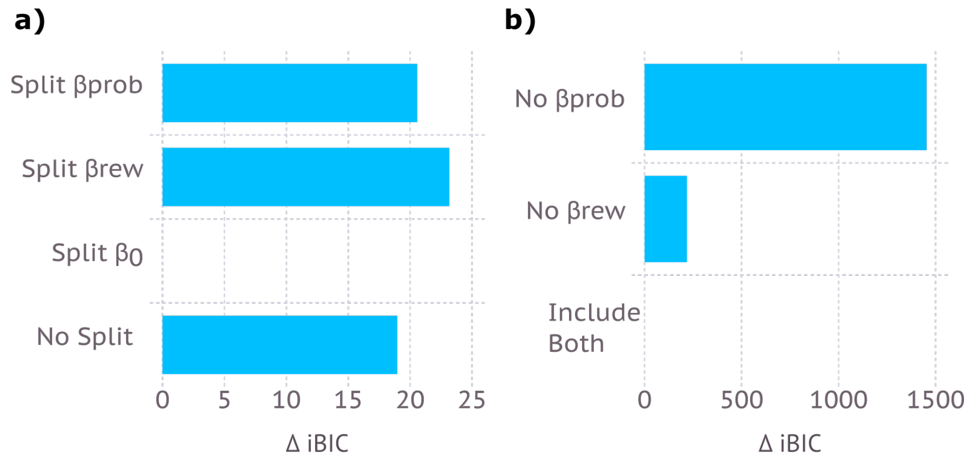

Supplementary Figure 2. Comparing variations of Additive Heuristic Model. A) Model that splits just β_0 between gain and loss trials fits provide the best account to choice data. "No Split" model is the Additive Heuristic model (as in Fig. 2c), yet does not use separate β_0 for gain and loss trials. "Split β_0" is the Additive Heuristic model as presented in Fig. 2c. "Split β_{rew}" and "Split β_{rew}" respectively include either a separate β_{rew} or β_{prob} parameter for gain and loss trials**. B) Models that did not use either probability information, "No β_{prob}", or did not use reward information "No β_{rew}" fit the data worse than the model that use both components, "Include Both".** b,c) Models are compare using integrated Bayesian Information Criterion (iBIC. Plots show iBIC relative to best fitting model (Additive Heuristic, Fig. 2c). A,B) iBIC reflects models fit to N=19 participants

Supplementary Note 1

Justification for use of Additive Heuristic Model to Parameterize Heuristic Use of Reward and Probability Information

The central goal of our neural analysis was to assess whether heuristic, idiosyncratic, reliance on probability and reward information in choice reflected differing strategies for which outcome' representations were reactivated during choice. This analysis required a participant-level parameterization of idiosyncratic, heuristic, use of reward and probability information in choice, as demonstrated in their behavior. Importantly, such treatment of reward and probability information is not a property of a single model, but rather a source of choice variance that can be captured by a variety of models. For example, whereas prospect theory allows for idiosyncratic treatment of probability information through individual variation in a probability weighting function, the additive heuristic model allows this through individual variation in a behavioral probability weight.

We conducted two analyses to determine which model provided the most useful parameterization for the purpose of guiding our neural analysis. First, we determined which model provided the best explanation of participants choices, using standard approaches to model comparison. Second, a model parameterization is only useful for indexing a choice strategy if its parameters can be identified in the task. Thus, in a second analysis, we compared the identifiability of the key parameters for each model.

Model comparison. We compared the ability of several models to explain participants choices. For the purposes of parameterizing idiosyncratic use of reward and probability information, the key models of interest were the additive heuristic model and prospect theory (Kahneman & Tversky, 1979) models, which both provide parameters for heuristic use of either type of information. Additionally, we also considered two recently proposed models based on sampling outcomes according to some probability distribution. Although these models do not explicitly have parameterization of heuristic use of reward and probability information, they do make claims about how outcome reactivations might relate to choices, and on this basis are potentially of interest for guiding MEG analysis. Finally, to compare any model to optimal weighting of reward and probability and reward information, we also compared each model to models which decided based on expected value.

Supplementary Figure 3 shows the goodness of fit of each model to participant's choices, as measured by computing the integrated Bayesian Information Criterion (iBIC) over the entire group of subjects for each model. For this analysis, all models were fit by maximizing the likelihood of choices, jointly with group-level distributions over the entire population using an Expectation Maximization (EM) procedure (Huys et al., 2011). This revealed the additive heuristic model provided the best fit to participants behavior ($\Delta \mathrm{iBIC}$ Additive Heuristic Model vs Prospect Theory = 142; Supplementary Fig. 3, left). In addition to the choice data from the MEG decision study, we additionally fit models to data from the additional priming study that was performed online. This again revealed the Additive Heuristic model provided a better fit to the data than a Prospect theory model ($\Delta \mathrm{iBIC}$ Additive Heuristic Model vs Prospect Theory = 946 Supplementary Fig. 3, right)

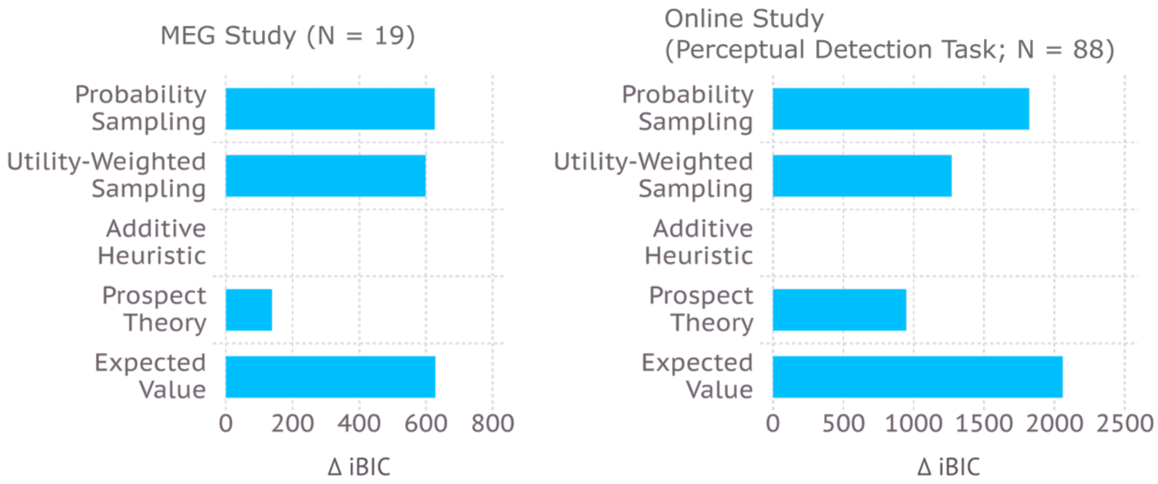

Supplementary Figure 3: Comparison of model iBIC scores. Each bar gives iBIC (integrated Bayesian Information Criterion) relative to the best fitting model. Left: Results of model-comparison to behavioral data from MEG study. N=19 participants. Right: Results of model-comparison from online priming/perceptual discrimination task data (Fig. 6) N = 88 participants. For both tasks, the best-fitting model was the additive heuristic model followed by a prospect theory model.

In addition to computing iBIC, which provide an overall measure of model fit to all participants, we also sought to obtain unbiased individual-level participant fits, such that we could perform a hypothesis test for whether, at the group level, the additive heuristic model provided a better fit than prospect theory. Thus, for each model, for each participant, we also computed unbiased per-participant log likelihoods, fitting each model in a flat manner, without a prior. Note that because the heuristic model and prospect theory share the same number of parameters, these per-subject log-likelihoods could be directly compared. Comparison of these unbiased log-likelihoods revealed a significant advantage for Additive Heuristic model compared to Prospect Theory both for the MEG as well as the online study: (MEG study: mean +/- sem log likelihood difference = 8.40 +/- 3.97, t(18) = 3.98, p = .048, two-sided paired sample t-test. Online Study: mean +/- sem log likelihood difference = 7.77 +/- 1.164, t(87) = 3.98, p < 1e-9).

Parameter Identifiability. In addition to comparing the ability of each model to explain participant's choices, we sought to examine the identifiability of key parameters governing heuristic use of probability and reward information for the two best performing models: the Additive Heuristic Model and Prospect Theory. To do so we first fit the two models to participants behavior using an EM approach, so as to identify group-level distributions of each parameter (parameterized as normal distributions with means and variances). Then, for each model, we generated 100 new datasets (with 19 participants each), repeatedly sampling parameters for each participant from the group level distributions. For each new dataset, we re-fit both models, again using the EM approach, in order to obtain parameter estimates for each participant. We then computed Pearson correlation coefficients comparing the true parameter values (used for each participant's the simulated data) with the recovered values. Supplementary Table 1, Pearson coefficients for prospect theory parameters, shows the key prospect theory parameters governing heuristic use of reward and probability information (γ and α,) had poor reliability (r = .64 and .43 for γ and α respectively). In contrast, Supplementary Table 2, Pearson coefficients for the Additive Heuristic model show that the key parameters governing heuristic use of reward and probability information (β_{prob} and β_{reward} respectively) had substantially better identifiability (r = .97 and .95 respectively).

Recovered Parameters					
True Parameters		β	γ	δ	α
	β	**.54**	.26	.04	.00
	γ	.36	**.64**	.01	-.04
	δ	-.05	-.01	**.62**	.51
	α	.63	.37	.27	**.43**

Supplementary Table 1: Recoverability of prospect theory parameters. Each cell shows Pearson correlation coefficient comparing simulated (true) parameters to best fit parameters across simulated participants.

Recovered Parameters					
True Parameters		β_{gain}	β_{loss}	β_{prob}	β_{reward}
	β_{gain}	**.93**	.03	.00	.00
	β_{loss}	.03	**.91**	-.02	-.02
	β_{prob}	.02	-.02	**.97**	-.05
	β_{reward}	-0.01	-.03	.06	**.95**

Supplementary Table 2: Recoverability of additive heuristic parameters. Each cell shows Pearson correlation coefficient comparing simulated (true) parameters to best fit parameters across simulated participants.

Taken together, a better performance in explaining participants choices, and a substantially better parameter identifiability, support the use of the additive heuristic model in parameterizing participant’s use of reward and probability information in choice.

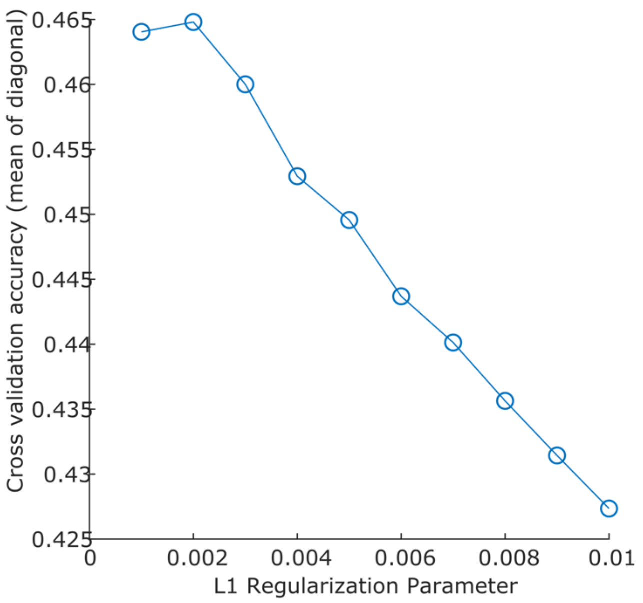

Supplementary Figure 4. Selection of classifier hyperparameter. Mean cross validation accuracy , across train/test-time points from 0-500ms (diagonal of Fig. 3c), across participants. We selected the L1 regularization parameter that maximized this score. Means are over N=19 participants.

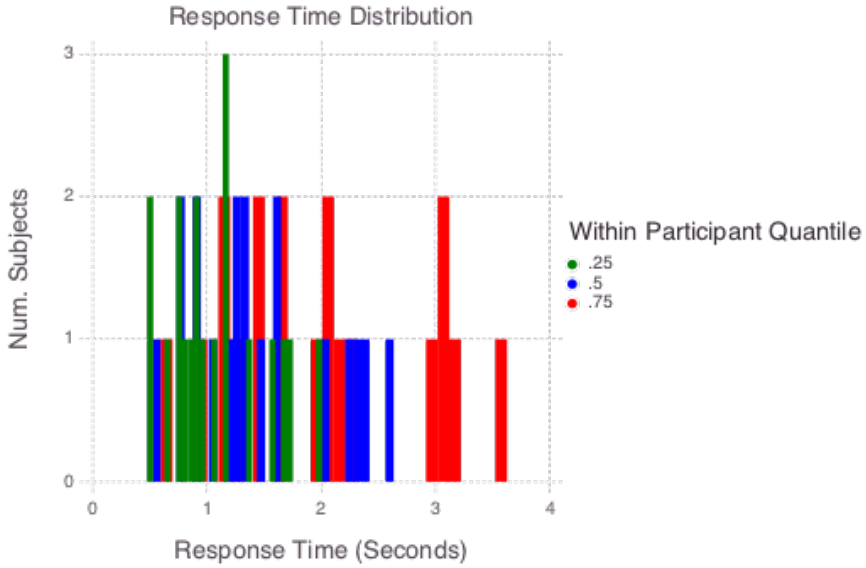

Supplementary Figure 5. Distribution of participant response time quantiles. Each color designates a different within participant response time quantile. Bar heights show the number of participants at that quantile. This is out of N=19 participants.

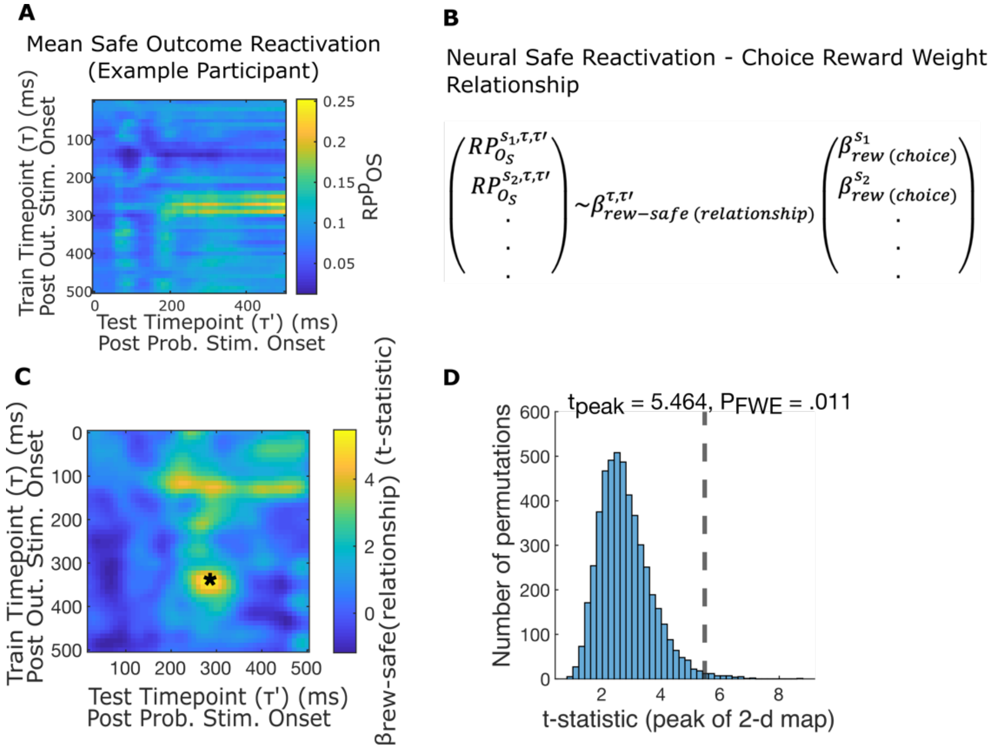

Supplementary Figure 6. Behavioral sensitivity to reward information relates to greater reactivation of safe outcome representation. In order to measure a tendency to reactivate a safe outcome representation, we computed the mean reactivation probability of the safe outcome representation, $\mathrm{RP}_{\mathrm{O_S}}^{s,\tau,\tau'}$ for participant, s, train timepoint, τ, following outcome stimulus onset and test timepoint, τ', following probability stimulus onset. **A) Safe Reactivation for Example Participant.** Image denotes safe reactivation probability, $\mathrm{RP}_{\mathrm{O_S}}^{s,\tau,\tau'}$, averaged across trials, for each train and task time-point, τ and τ', for an example participant, s. **B) Measuring Relationship Between Safe Outcome Reactivation and Behavioral Reward Integration** In order to measure the between-participant relationship between reactivation of the safe outcome and behavioral integration of reward information into choice, as measured by the reward component of the additive heuristic model ($\beta^{s}_{\mathrm{rew\ (behavior)}}$), we regressed $\beta^{s}_{\mathrm{rew\ (behavior)}}$ onto between participant measure of mean safe reactivation, $\mathrm{RP}_{\mathrm{O_S}}^{s,\tau,\tau'}$ separately for each , τ and τ'. **C-D) Safe Outcome Reactivation Relates to Behavioral Sensitivity to Reward Information.** c) Image shows a t-statistic for this regression (applied to 19 participants), for each train and task timebin, smoothed with a Gaussian kernel (σ = 1.5 timebins). *: $\mathrm{P_{FWE}}$ = .011, non-parametric permutation test on image peak. d) Histogram shows null distribution of maximum t-statistics over 5000 2-d maps, each generated by randomly shuffling $\beta^{s}_{\mathrm{rew\ (behavior)}}$ between participants. Dashed line shows true maximum t-statistic. T statistics reflect regressions over N=19 particpants.

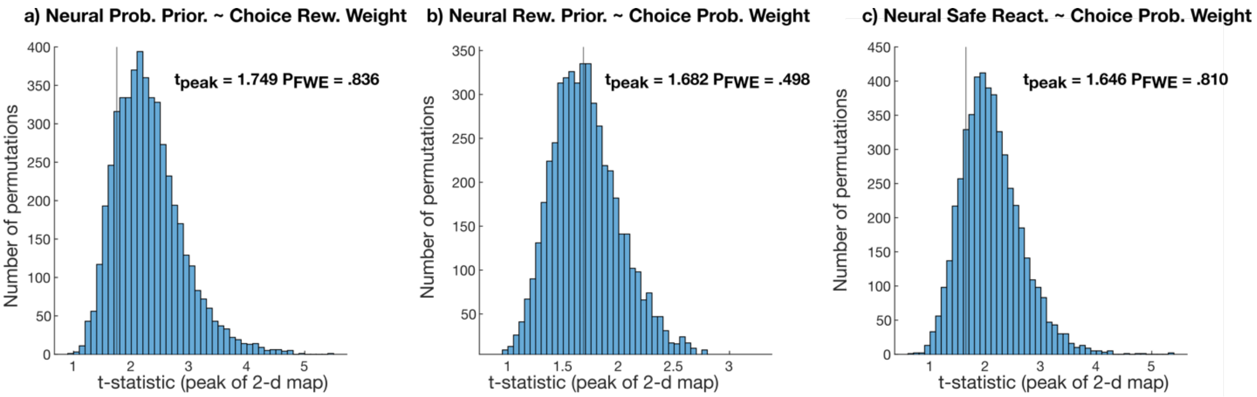

Supplementary Figure 7. Relationships between MEG outcome reactivation and alternative choice weights. A) We did not identify a positive relationship between neural probability prioritization and behavioral reward weight. B) We also did not identify a positive relationship between neural reward prioritization and behavioral probability weight. c) We also did not identify a positive relationship between neural safe reactivation and behavioral probability weight. T-statistics reflect regressions over N=19 participants.

Supplementary Note 2

Estimating Behavioral-Neural Correlations

A central challenge with estimating effect sizes of relationships (e.g. r) between behavioral and neural measures (e.g. Neural Probability Prioritization versus Behavioral Probability Weight) is selecting a train and test time-point at which to measure the relationship. Whereas the significance of the relationship in general can be assessed by selecting the max t-statistic over the 2-d map of train and test time-points and comparing this to a null distribution of max-t statistics (generated from shuffled assignments of behavioral parameters to participants), this approach cannot be used to identify an unbiased effect size of the relationship. Notably, the effect-size at the maximum train and test time-point is biased due to the selection process. One solution here is to use a set of held-out participants in order to select a time-point (by selecting the train and test time-points which maximize the relationship), and use this held out set to evaluate the effect size at that time-point. However, using only a portion of the data to identify a time-point risks misidentifying the train and test time-points at which the relationship occurs, and necessarily uses less data to evaluate the relationship.

Consequently, we used a variant of this approach, with the aim of making maximal use of our available data. For each participant, j, we selected a time-point using all participants except j, taking the time-point which maximized the relationship. We then took the neural measure from participant j at that train and test time-point. Repeating this, treating each participant as the held-out participant, provided for each participant an unbiased held-out estimate of the neural effect. We then evaluated the Pearson correlation coefficient between these between-participant held-out, unbiased, neural measurements and the between-participant behavioral measurements.

We implemented a simulation to ensure this approach does not produce biased correlation values. For each iteration of the simulation, we generated 19 (one for each participant) behavioral and neural measurements with some true correlation (by sampling from a multi-variate normal distribution). These neural measurements were then embedded into a matrix with one column for each time-point (Number-of-Time-Points=10 X 19 participants), where the other columns corresponding to alternative time-points comprised neural measurements generated to be uncorrelated with the behavioral measurements. We then estimated the correlation using the above described procedure of estimating a time-point separately for each held-out participant (using the non-held out participants) and evaluating the neural measurement for that held-out participant at the selected time-point. Supplementary Fig. 8 shows the findings, for each true correlation coefficient (true r), based upon repeating this process 1000 times and taking either the mean or median estimated correlation value. As apparent from the plot, the estimated correlations are not inflated, and in fact are biased toward lower values, due the procedure frequently failing to select the appropriate time-point at which to assess participant’s measurements.

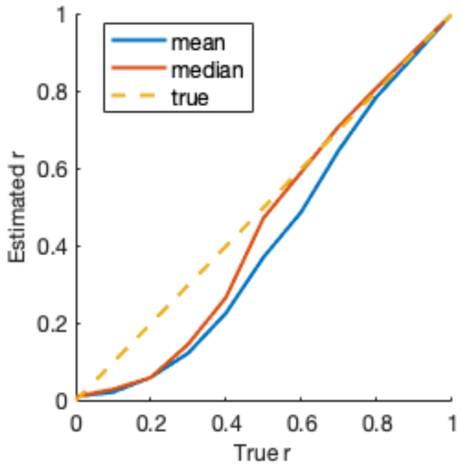

Supplementary Figure 8. Results of simulating procedure for estimating correlations by repeatedly using all participants but one to select a time-point.

Supplementary Figure 9 shows the results of applying this procedure to the four behavioral-neural relationships presented in the main text. We found that this procedure generated strong correlation values for all the relationships, except for the relationship between Behavioral Reward Weight and Neural Safe Activation. We think this is because this relationship, in the main-text, is strongly influenced by a single participant who has a high Behavioral Reward Weight, and also large Neural Safe Activation at a particular train and test-time-point. However, when this participant is unable to contribute to time-point selection, their Safe Activation is taken at a different activation at which it is not high.

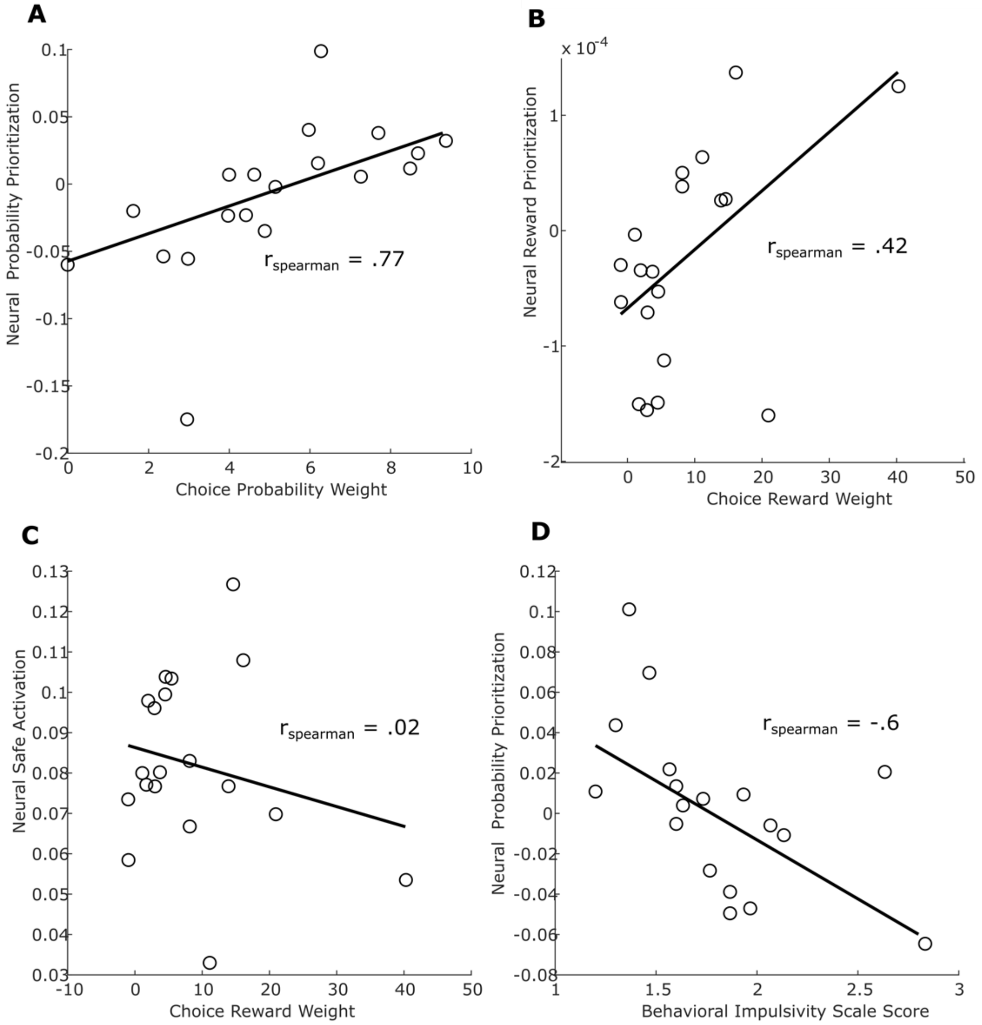

Supplementary Figure 9. Correlations derived from unbiased estimation process. A. Relationship between Neural Probability Prioritization and Behavioral Probability weight. B. Relationship between Neural Reward Prioritization and Behavioral Reward Weight. C. Relationship between Neural Safe Activation and Behavioral Reward Weight. D. Relationship between Neural Probability Prioritization and Behavioral Impulsivity Scale (BIS) Score. N=19 participants.

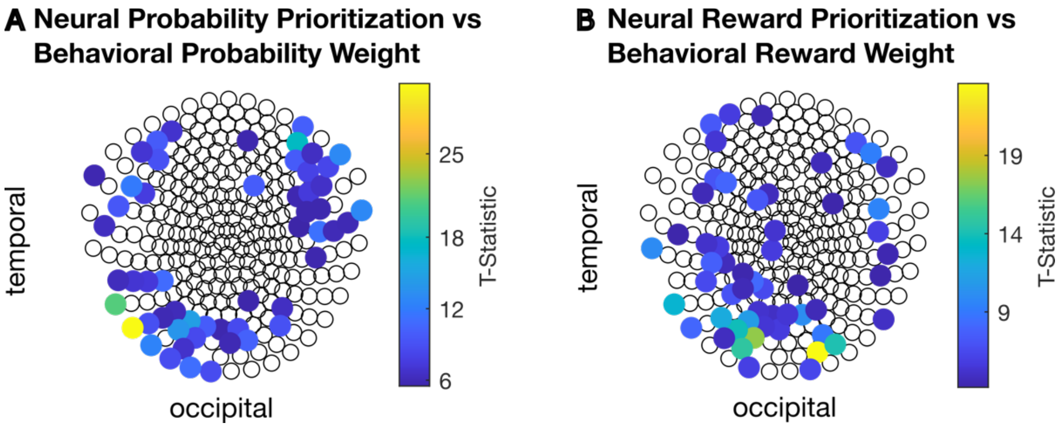

Supplementary Figure 10. Contribution of sensors toward key behavioral neural relationships. To explore which brain areas contributed toward the key behavioral-neural effects reported, we repeated our key analysis measuring the relationships between neural probability prioritization and behavioral probability weights and neural reward prioritization and behavioral reward weights 5000 times, each time using only a subset of 50 randomly selected (per-iteration) sensors. To determine the importance of each sensor for either relationship, we performed a regression, estimating the t-statistic of either effect as a function of whether a given sensor was included in the analysis. We take the t-statistic of a given sensor in this regression to reflect a weight of that sensor's importance toward either effect. **A.** Key sensors for relationship between Neural Probability Prioritization and Behavioral Probability Weight. **B.** Key sensors for relationship between Neural Reward Prioritization and Behavioral Reward Weight. For both A and B, for visualization proposes we only show T-Statistics for sensors with T-Statistics in the top 20 percentile. Both relationships were largely driven by occipital and temporal cortices, with a small number of frontal sensors involved as well. There was a small but significant correlation between the t-statistics over sensors for either effect $(r(270) = .13, P = .029)$ demonstrating that the sensors contributing to either effect are overlapping. Each map reflects t-statistic over N=19 participants.

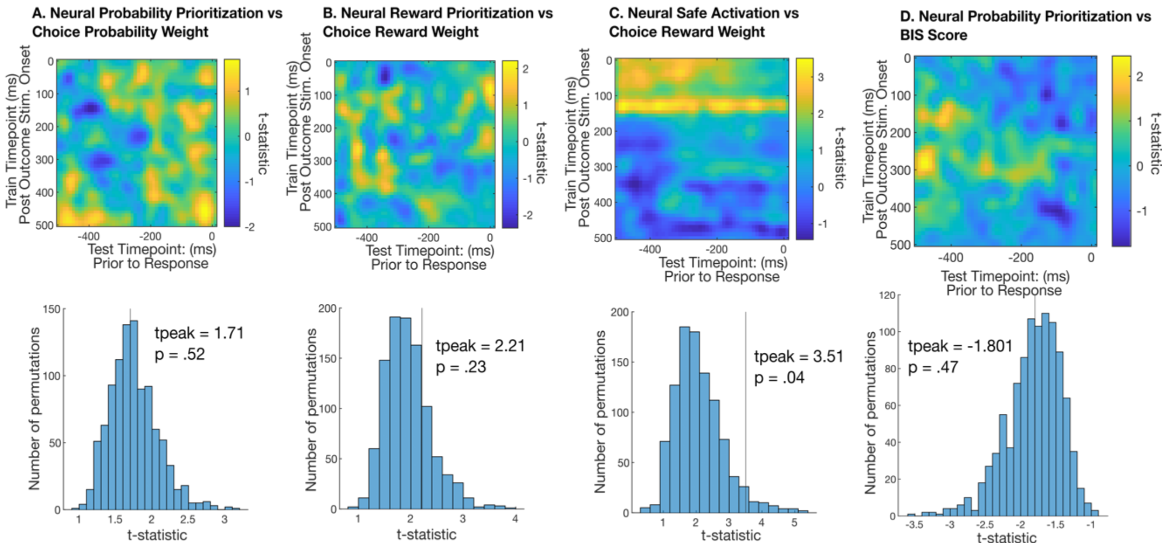

Supplementary Figure 11. Analysis of key effects locked to response time. When locked to the time of response, we only observed a relationship between Neural Safe Activation and Choice Reward Weight. This suggests that key reactivation events occurred in a manner more locked to presentation of the Probability stimulus, rather than to the response. A) Relationship between Choice Probability Weight and Neural Probability Prioritization. B) Relationship between Choice Reward Weight and Neural Reward Prioritization. C) Relationship between Choice Reward Weight and Neural Safe Activation. D) Relationship between BIS scores and Neural Probability Prioritization. T statistics reflect regressions over N=19 participants.

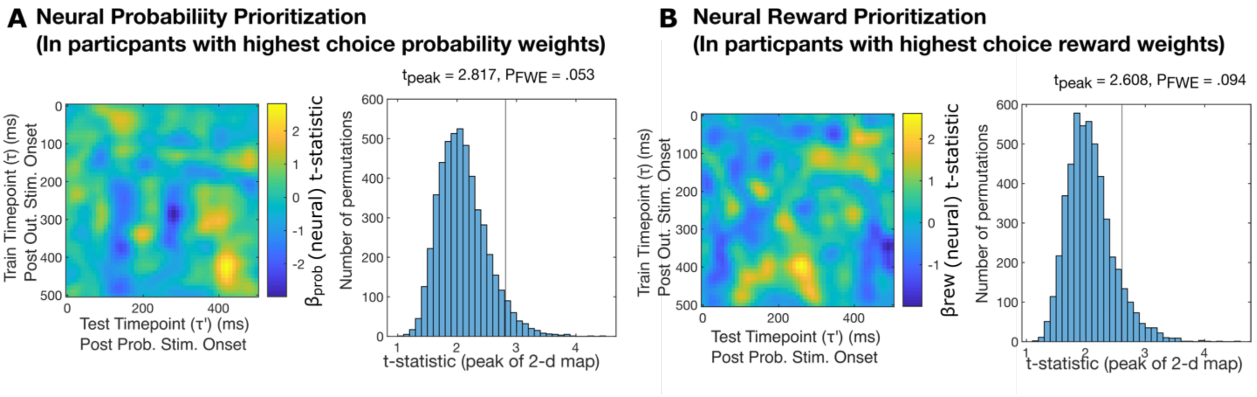

Supplementary Figure 12. Examining reactivation in participants with highest Choice Probability Weights (A) and highest Choice Reward Weights (B). A) Neural Probability Prioritization in participants with highest 33 percentile Choice Probability Weights (N = 7 participants). B) Neural Reward Prioritization in participants with highest 33 percentile Behavioral Reward Weights (N = 7 participants).

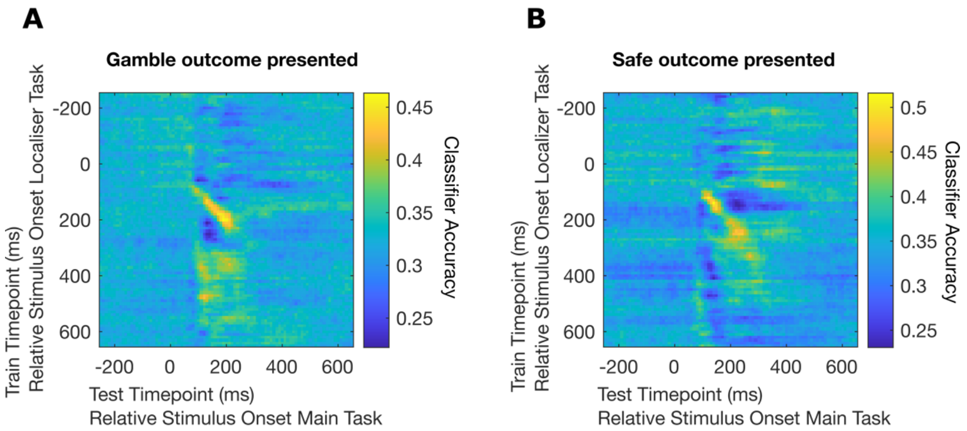

Supplementary Figure 13. Classification accuracy when training on localizer task and testing on outcomes presented in the decision making task. A) Accuracy (out of three outcome stimuli) when the true outcome stimulus is a gamble outcome. B) Accuracy (out of three outcome stimuli) when the true outcome stimulus is the safe outcome. Means are over N=19 participants.

	Mean (per run)	Stand. Dev (per run)
Total iCs removed	12.428	2.5429
Removed for Kurtosis	9.214	1.3966
Removed for Correlation with EOG	2.7045	1.3966

Supplementary Table 3: Mean number of ICA components removed, for either kurotosis or correlation with EOG.

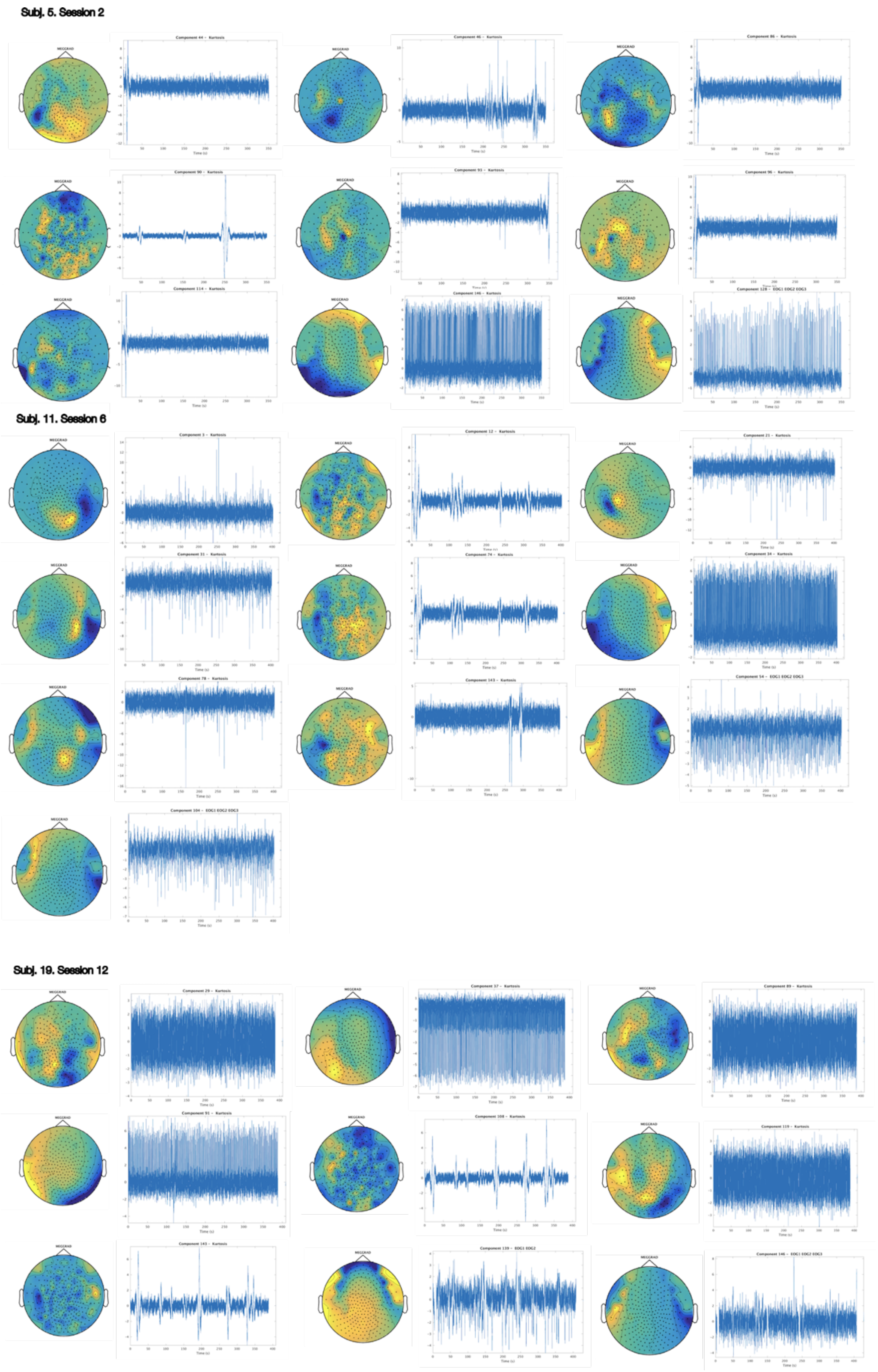

Supplementary Figure 14. Representative ICA components removed for three participants.

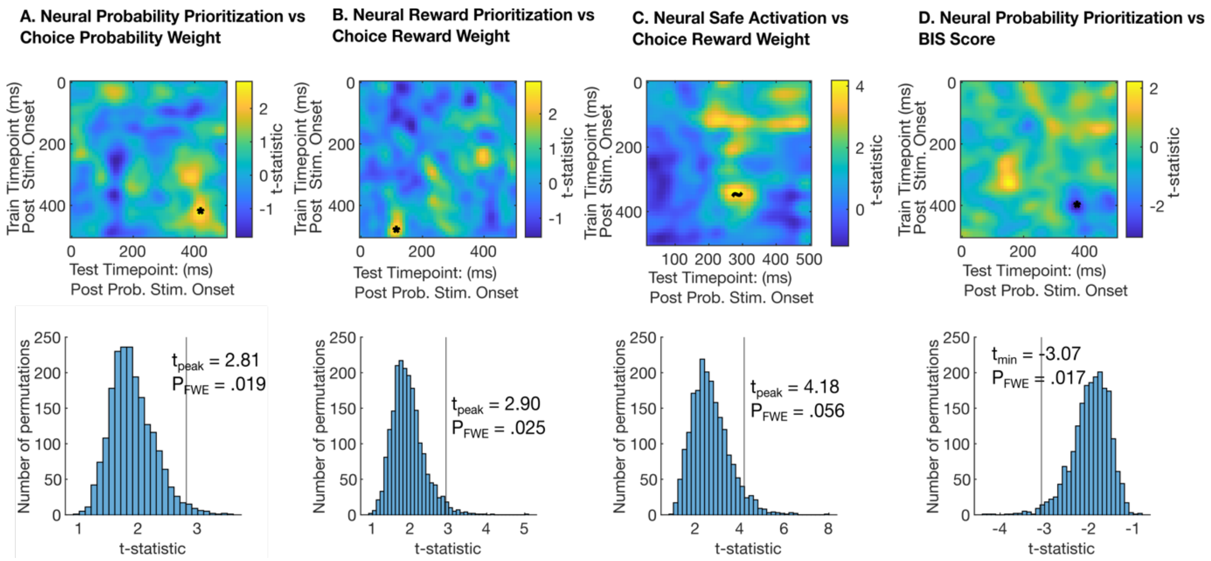

Supplementary Figure 15. Effect of between session change in decoding accuracy control covariate on all behavioral-neural relationships. The decision-making task took place across 8 scanning sessions. To assess whether changes in decoding accuracy, perhaps due to between session movement, affected our results, we estimated for each participant a measure of how much our ability to decode visual stimuli decreased across runs. For each participant and run, we computed the outcome decoding accuracy at each train time (on localizer task image) and test time (on decision task outcome image), varying each between 0 and 500 ms, and took the maximum accuracy over this smoothed 2-D image (with $\sigma = 1.5$). Fitting a linear mixed effects model to predict this accuracy measure as a function of run, with participant identity included as a random effect, revealed that decoding accuracy indeed slightly decreased over runs ($\beta_{\mathrm{accuracy-change-per-run}} = -.004,\ t(19) = -11.99, P = .003$). To see whether this change in decoding accuracy across sessions affected our results, we estimated the extent to which each individual participant decreased decoding accuracy across runs as the per-participant random effect from this model, and included this as a between-participant control covariate in each behavioral-relationship. Including this measure as a control covariate did not change any of our results. **A.** Relationship between Neural Probability Prioritization and Choice Probability Weight. **B.** Relationship between Neural Reward Prioritization and Choice Reward Weight. **C.** Relationship between Neural Safe Activation and Choice Reward Weight. **D.** Relationship between Neural Probability Prioritization and BIS Score. T-statistics reflect regressions over N=19 participants.

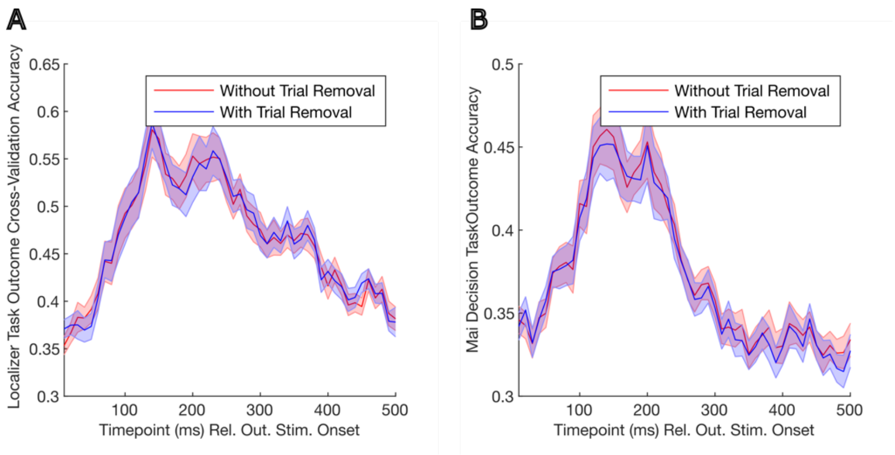

Supplementary Figure 16: Effect of trial removal on decoding accuracy. Overall, trial removal did not affect decoding accuracy. **A.** Decoding accuracy for held-out localizer data with and without trial removal. **B.** Decoding accuracy for task outcome images with and without both localizer and task trial removal. Lines reflect means over N=19 participants.

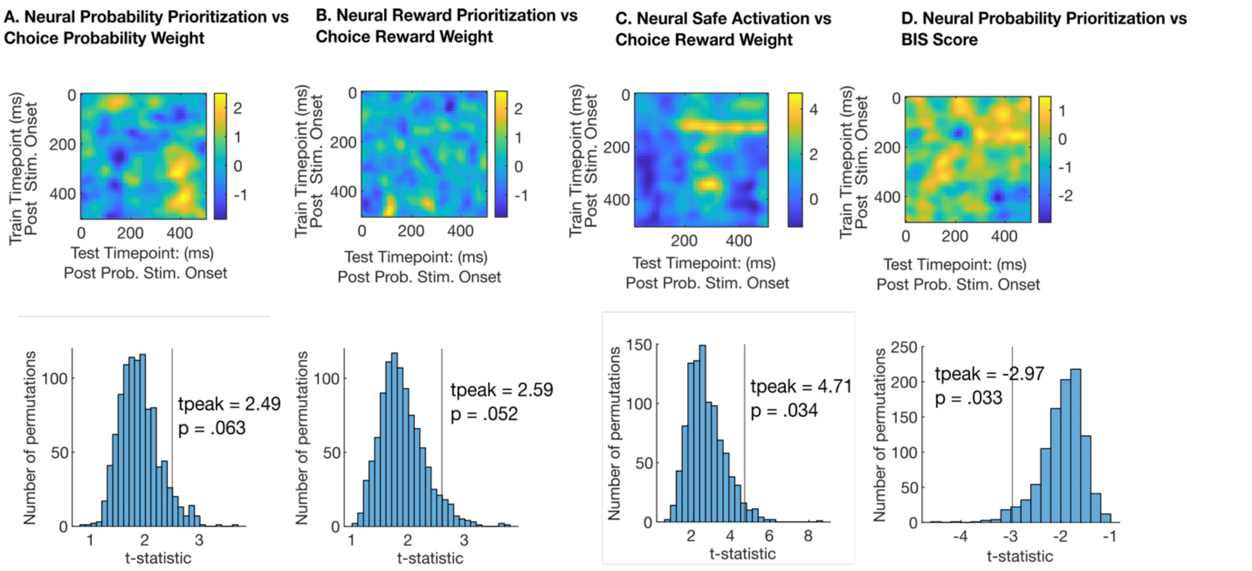

Supplementary Figure 17: Effect of trial removal on all behavioral-neural relationships. Overall, trial removal marginally weakens the strength of results, but does not change them qualitatively. **A.** Relationship between Neural Probability Prioritization and Choice Probability Weight. **B.** Relationship between Neural Reward Prioritization and Choice Reward Weight. **C.** Relationship between Neural Safe Activation and Choice Reward Weight. **D.** Relationship between Neural Probability Prioritization and BIS Score. N=19 participants.
