# Supplementary material for: Heuristics in risky decision-making relate to preferential representation of information
Source: Nat Commun. 2024 May 20;15:4269. doi: 10.1038/s41467-024-48547-z (PMC11106265; doi:10.1038/s41467-024-48547-z)
Supplement: Supplementary file 3 — Reporting Summary [file 41467_2024_48547_MOESM3_ESM.pdf]

nature portfolio

Corresponding author(s): Evan Russek

Last updated by author(s): Apr 9, 2024

Reporting Summary

Nature Portfolio wishes to improve the reproducibility of the work that we publish. This form provides structure for consistency and transparency in reporting. For further information on Nature Portfolio policies, see our Editorial Policies and the Editorial Policy Checklist.

Statistics

For all statistical analyses, confirm that the following items are present in the figure legend, table legend, main text, or Methods section.

n/a	Confirmed	
☐	☒	The exact sample size (n) for each experimental group/condition, given as a discrete number and unit of measurement
☐	☒	A statement on whether measurements were taken from distinct samples or whether the same sample was measured repeatedly
☐	☒	The statistical test(s) used AND whether they are one- or two-sided<br>*Only common tests should be described solely by name; describe more complex techniques in the Methods section.*
☐	☒	A description of all covariates tested
☐	☒	A description of any assumptions or corrections, such as tests of normality and adjustment for multiple comparisons
☐	☒	A full description of the statistical parameters including central tendency (e.g. means) or other basic estimates (e.g. regression coefficient) AND variation (e.g. standard deviation) or associated estimates of uncertainty (e.g. confidence intervals)
☐	☒	For null hypothesis testing, the test statistic (e.g. F, t, r) with confidence intervals, effect sizes, degrees of freedom and P value noted<br>*Give P values as exact values whenever suitable.*
☒	☐	For Bayesian analysis, information on the choice of priors and Markov chain Monte Carlo settings
☒	☐	For hierarchical and complex designs, identification of the appropriate level for tests and full reporting of outcomes
☐	☒	Estimates of effect sizes (e.g. Cohen's d, Pearson's r), indicating how they were calculated

Our web collection on statistics for biologists contains articles on many of the points above.

Software and code

Policy information about availability of computer code

Data collection	Experiment presented using custom Javascript code and jspsych (version 5.0.3).
Data analysis	Behavioral analysis: Julia programming language (version 1.5). MEG analysis: Matlab (version 2019a). MEG preprocessing: OSL (https://ohba-analysis.github.io/osl-docs/).

For manuscripts utilizing custom algorithms or software that are central to the research but not yet described in published literature, software must be made available to editors and reviewers. We strongly encourage code deposition in a community repository (e.g. GitHub). See the Nature Portfolio guidelines for submitting code & software for further information.

Data

Policy information about availability of data

All manuscripts must include a data availability statement. This statement should provide the following information, where applicable:

- Accession codes, unique identifiers, or web links for publicly available datasets
- A description of any restrictions on data availability
- For clinical datasets or third party data, please ensure that the statement adheres to our policy

Raw and preprocessed MEG data have been deposited in the OpenNeuro database57, under accession code doi:10.18112/openneuro.ds005065.v1.0.0. Behavioral data for both tasks is deposited at zenodo58, https://doi.org/10.5281/zenodo.10950132.

Research involving human participants, their data, or biological material

Policy information about studies with human participants or human data. See also policy information about sex, gender (identity/presentation), and sexual orientation and race, ethnicity and racism.

Reporting on sex and gender	Our sample is well balanced for sex (as reported by the participants). We do not collect information pertaining to gender.
Reporting on race, ethnicity, or other socially relevant groupings	Participants are not grouped based on race, ethnicity or other socially relevant groupings. We do not expect that these variables would impact our results.
Population characteristics	For the MEG study, Of the 21 recruited participants, 13 were female. The mean age was 23.67 with a range of 18 to 36. For the perceptual detection task, of the 100 participants recruited, mean age was 27.6, 35 were female.
Recruitment	For the MEG study, participants were recruited from the University College London Institute of Cognitive Neuroscience participant database. For the perceptual detection task, participants were recruited on Prolific. While both of these recruitments may be biased toward speficific populations (either university students or prolific users) the consistent results across both studies provides some evidence that these self-selection biases do not impact the results.
Ethics oversight	University College London Research Ethics Committee. Participants in both studies provided informed consent.

Note that full information on the approval of the study protocol must also be provided in the manuscript.

Field-specific reporting

Please select the one below that is the best fit for your research. If you are not sure, read the appropriate sections before making your selection.

☒ Life sciences ☐ Behavioural & social sciences ☐ Ecological, evolutionary & environmental sciences

For a reference copy of the document with all sections, see nature.com/documents/nr-reporting-summary-flat.pdf

Life sciences study design

All studies must disclose on these points even when the disclosure is negative.

Sample size	For the MEG study, we recruited 21 participants from University College London subject databases who provided informed consent prior to beginning the study. Based on consideration from prior literature, we chose a sample of 30 participants, however, due to the coronavirus pandemic and the UK lockdown, we were required to stop collecting data at 21 participants. Although this number of participants is less than intended, we note that it is within the range for similar studies in the field (Doll et al., 2015; Momennejad, Otto, Daw, & Norman, 2018; Wimmer & Shohamy, 2012, Park et al., 2021).<br><br>For the perceptual detection study, we recruited 100 participants. No sample size calculation was performed, however this number is far greater than the number of participants used in similar studies measuring representation through response time (Garvert et al., 2017, Bornsein and Daw, 2013).
Data exclusions	For the MEG study, two participants were removed from analysis for choosing the same action on greater than 80% of trials , thus leaving 19 participants included in the main analysis (Figs. 2 - 5). We additionally failed to collect questionnaire data for one participant. Thus the neural-questionnaire analysis (Fig. 7) reflects data from 18 participants.<br><br>For the perceptual detection study, using the same criteria, we removed 5 participants. An additional 4 participants were removed due to failure to make any responses in the perceptual detection trials. Finally, data from 3 participants were lost due to errors in recording. This left 88 participants for analysis.
Replication	Due to logistical constraints (covid-19 pandemic and the lead author moving to an institution in the United States), no explicit replication tests were performed, though the behavioral perceptual detection study presented in Fig. 6 presents a conceptual replication of the MEG study.
Randomization	Assignment of which visual stimuli were assigned to which choices and outcomes was randomized accross participants. Order of trials and gain and loss blocks were also randomized.
Blinding	Full information was given to participants and there was no treatment; thus blinding is not applicable. Specifically, there were not different condition groups, thus there was no information from which to blind participants. However, participants were not informed of the purpose of the study until after they complete the task.

Reporting for specific materials, systems and methods

We require information from authors about some types of materials, experimental systems and methods used in many studies. Here, indicate whether each material, system or method listed is relevant to your study. If you are not sure if a list item applies to your research, read the appropriate section before selecting a response.

Materials & experimental systems

n/a	Involved in the study
☒	☐ Antibodies
☒	☐ Eukaryotic cell lines
☒	☐ Palaeontology and archaeology
☒	☐ Animals and other organisms
☒	☐ Clinical data
☒	☐ Dual use research of concern
☒	☐ Plants

Methods

n/a	Involved in the study
☒	☐ ChIP-seq
☒	☐ Flow cytometry
☒	☐ MRI-based neuroimaging

Plants

Seed stocks	N/A
Novel plant genotypes	N/A
Authentication	N/A
